# Is There a Potential Link between Gastroesophageal Reflux Disease and Recurrent Respiratory Tract Infections in Children?

**DOI:** 10.3390/diagnostics13132310

**Published:** 2023-07-07

**Authors:** Vasile Valeriu Lupu, Gabriela Stefanescu, Ana Maria Laura Buga, Lorenza Forna, Elena Tarca, Iuliana Magdalena Starcea, Cristina Maria Mihai, Laura Florescu, Andrei Tudor Cernomaz, Adriana Mocanu, Viorel Tarca, Aye Aung Thet, Ancuta Lupu

**Affiliations:** 1 Pediatrics, “Grigore T. Popa” University of Medicine and Pharmacy, 700115 Iasi, Romania; 2 Gastroenterology, “Grigore T. Popa” University of Medicine and Pharmacy, 700115 Iasi, Romania; 3 Department of Surgery II—Pediatric Surgery, “Grigore T. Popa” University of Medicine and Pharmacy, 700115 Iasi, Romania; 4 Pediatrics, Faculty of General Medicine, Ovidius University, 900470 Constanta, Romania; cristina2603@yahoo.com; 5 Mother and Child Medicine Department, “Grigore T. Popa” University of Medicine and Pharmacy, 700115 Iasi, Romania; 6 3rd Medical Department, “Grigore T. Popa” University of Medicine and Pharmacy, 700115 Iasi, Romania; 7 Department of Preventive Medicine and Interdisciplinarity, “Grigore T. Popa” University of Medicine and Pharmacy, 700115 Iasi, Romania; viorel.tarca@umfiasi.ro; 8 Faculty of General Medicine, “Grigore T. Popa” University of Medicine and Pharmacy, 700115 Iasi, Romania

**Keywords:** recurrent respiratory tract infections, gastroesophageal reflux disease, children, Boix-Ochoa score, pH-metry

## Abstract

Background: The implications of gastroesophageal reflux disease in respiratory tract infections have been investigated over time. The aim of our study was to evaluate the relationship between these two pathologic entities and the outcome after proper antireflux treatment. Methods: A group of 53 children with recurrent respiratory tract infections admitted in the gastroenterology clinic of a children’s hospital in North-East Romania was investigated for gastroesophageal reflux disease through 24 h pH-metry. Those with a Boix-Ochoa score higher than 11.99 received proton pump inhibitor treatment and were reevaluated after 2 months. Results: A total of 41 children were found with a positive Boix-Ochoa score. After 2 months of antireflux therapy, eight patients still had a positive Boix-Ochoa score. Conclusions: Recurrent respiratory tract infections with symptoms resistant to treatment should be considered a reason to investigate for gastroesophageal reflux, because the symptoms may be due to micro- or macro-aspiration of the gastric refluxate or to an esophageal-bronchial reflex mediated through the vagal nerve.

## 1. Introduction

Gastroesophageal reflux becomes a disease when the physiologic retrograde passage of gastric content due to transient lower esophageal sphincter relaxation crosses a biologic threshold and determines disturbing symptoms and complications [1].

In children, unlike adults, there are some particular elements that intervene in the pathogenesis of GERD. These elements are characteristic for a certain age range. First of all, in infants younger than six months, reflux episodes are due to the physiological immaturity of the lower esophageal sphincter, the type of exclusively liquid feeding and the extended periods of time that the newborn and infants spend in a horizontal position. To all this are added elements related to the anatomical dynamic of the esophagus and stomach, by which we refer to the lower volumetric capacity of the esophagus and its shorter intraabdominal portion, as well as the relationship between the volume of food that a child at this age consumes relative to his body mass [2,3]. Beyond this age, the mechanisms through which GERD occurs tend to resemble those of adults.

The connection through which GERD can interfere with the functioning of the respiratory tract has its origins in embryonic life. Several events that take place during this stage of human evolution—the organ formation phase—represent the basis for the links between the digestive tract and the pathology of the respiratory tract. Due to the cross between the respiratory and digestive tracts in the pharynx, there are both anatomical and neural connections among these two pathways. Therefore, there are a series of reflexes at this level intended to protect the airways from the penetration of reflux liquid: the pharyngeal swallow reflex, the esophago-upper esophageal sphincter contractile reflex and the esophago-glottal closure reflex. Also, the fact that on the laryngeal surface of the epiglottis there are “taste buds” similar to those present on the tongue, determines the closure of glottis when water and hydrochloric acid reach its surface, to keep away substances that should not enter the respiratory tract [4]. Respiratory symptoms such as cough, hoarseness and odynophagia can sometimes be the only signs of gastroesophageal reflux, many patients (especially pediatric ones) are lacking in typical reflux signs or symptoms [5].

Epidemiological data regarding pediatric gastroesophageal reflux disease (GERD) is limited; it is estimated that 1.8% to 8.2% of all children have GERD, with an incidence that decreases until the age of 1 and then rises again to a maximum at the age of 16 [6].

Manifestations that can occur due to GERD can be grouped into two large categories: esophageal and extraesophageal syndromes. The esophageal symptoms refer to those pathological entities related to the esophagus or the anatomical region where it is located, and the extraesophageal syndromes include mainly pathology of the respiratory tract as follows: reflux cough syndrome, reflux laryngitis syndrome, reflux asthma syndrome, reflux dental erosion syndrome, pharyngitis, sinusitis, idiopathic pulmonary fibrosis and recurrent otitis media [7].

Studies have already established a clear link between the esophageal syndromes listed above, but with regard to the other extraesophageal pathological entities—the recurrent respiratory tract infections—data from literature suggest that GER could be in fact one of the multiple factors that contribute to their occurrence and not a single etiological factor [8]. On the same note, when GERD does not manifest itself with the typically known symptoms of regurgitation or heartburn, it may be not suspected as a leading cause for respiratory disorder, and hence the treatment result may be unsatisfactory and the disease could be mistakenly considered refractory to treatment [9].

The correlation between gastroesophageal reflux and pulmonary pathology was first noted by Mendelson in 1887, who reported symptoms similar to those seen in asthmatic patients [5]. Since then, numerous studies have elucidated and strengthened the evidence provided that supports the link between a series of respiratory pathologies and the presence of gastroesophageal reflux. For example, asthma refractory to treatment was a challenge for clinicians until research revealed that in several studies more than a half of patients with asthma tested through continuous pH-metry presented with GERD [10,11]. Considering these new findings, the suspicion was raised that wheezing, although a symptom commonly seen in asthmatic patients, could also be linked to GERD, an extra-respiratory pathology that acts as a trigger factor [12,13]. Last but not least, the negative effects of acid reflux which can extend even to the level of the oral cavity should not be ignored. Caries is a public health issue, with GERD being a contributing factor to dental erosion, for patients of all ages as data in literature suggests [14,15,16].

The aim of the study presented in this paper was to determine the relationship between recurrent respiratory tract infections and GERD in pediatric patients aged 6 months to 162 months and to evaluate patients’ response after antireflux treatment.

## 2. Materials and Methods

The study took place in the gastroenterology clinic of a regional emergency hospital for children in North-East Romania that serves pediatric patients from seven surrounding counties. It is a retrospective study that involved 234 patients admitted to the clinic in a 5-year interval out of which we focused on those with recurrent respiratory tract infections. Informed consent was signed by all the patients’ caregivers at admission and approval from the hospital’s ethics committee was obtained.

Patients were diagnosed with GERD after being monitored for 24 h through continuous esophageal pH-metry and the diagnosis of respiratory pathology was established following anamnesis, objective clinical examination, laboratory tests and imaging explorations. Cases were selected for the study after they met the inclusion criteria listed in Table 1 and patients were excluded based on the criteria presented in the same table.

The pH measurement procedure involves a series of steps. First, the patient must stop the ingestion of both liquids and solids for a minimum of 6 h (if the patient is older than 12 months) or 3 h (if the patient is 12 months or less) prior to investigation. Use of medication known to modify the gastric secretion must also be stopped before the procedure within a certain timeframe specific to each drug (6 h for antacid drugs, 48 h for prokinetic drugs, 3 days for H2 receptor inhibitors and 7 days for proton pump inhibitors). The second step implies calibrating the device; this is done by performing two measurements in fluids with different pH levels of 1 and 7. After calibrating the device, the measurement can begin. In order to insert the electrode, the patient is seated in left lateral decubitus (for infants and small children) or in seated position (for children over the age of six). Then the lubricated electrode is inserted through one nostril until it reaches 5 cm above the lower esophageal sphincter. Next, the patient’s caregiver or the patient itself, depending on age, is advised to register symptoms and changes in body position (supine or standing) and to press the device’s button when these occur.

In order to monitor the esophageal pH, the Medtronic Digitrapper^®^ pH 100 SN 37660 with Zinetics 24 and ComforTEc by Sandhill multiuse catheters was used. The measurements were recorded using Polygram.Net^TM^ pH software (version 4.21). Physiological and pathological reflux episodes were differentiated by the pH values above, respectively, below 4. The Boix-Ochoa score was used to establish that the reflux episodes were pathological, cases scoring a value higher than 11.99 were included in the statistics [17,18,19].

To consider whether patients had recurrent respiratory tract infections, we used as a guide the next three criteria for defining recurrent respiratory tract infections: a minimum of 6 respiratory infections over the last 12 months; a minimum 1 episode of upper respiratory tract infection per month between September and April; a minimum 3 episodes of lower respiratory tract infection over the last 12 months [20]. In order to be included in the study, the patients had to check at least 1 of the 3 criteria mentioned above.

After being diagnosed with GERD, all patients received a two-month treatment course with PPIs (esomeprazole 1 mg/kg/day for children aged 1 month to 1 year, 20 mg/day for children with a weight below 55 kg, and 40 mg/day for those weighing over 55 kg) during which they came for the monthly check-up.

Data was processed using IBM SPSS Statistics 20 and correlation analysis was performed with Pearson parametric correlation. The correlation coefficients were established for a 95% confidence interval.

## 3. Results

**Figure 1:**
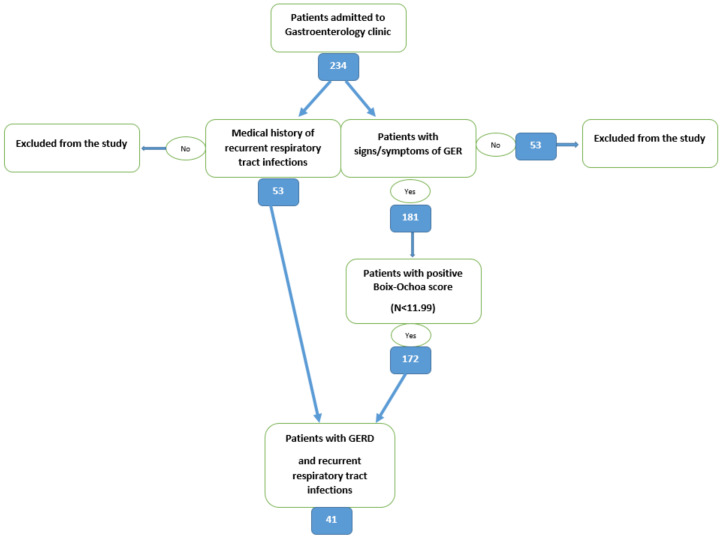
Flowchart of the selection process.

After establishing the diagnosis of GERD and history of recurrent respiratory tract infection and applying the inclusion and exclusion criteria, we found that of all 234 children, 53 (22.64%) patients had recurrent respiratory tract infections and 41 (77.36%) were associated with GERD (*p* = 0.0470, 95% confidence interval) (Figure 1).

Furthermore, we analyzed the number of cases that associated GERD and infectious respiratory pathology depending on the respiratory tract portion involved. We observed that 66% of all cases with respiratory tract infections were located above the larynx. Of these 35 cases, 27 (77.14%) were associated with GERD. A similar pattern was seen in children with lower respiratory tract infections and GERD, even if they were fewer in number (18 cases), the proportion was preserved, 77.7% were associated GERD (see Table 2). Data on gender distribution and place of living are presented in Table 3 and Table 4. We observed that there was a higher number of male patients with respiratory tract infections than female patients (39 vs. 14, with a *p* value of 0.038) and the same pattern was seen in patients that associated GERD and respiratory pathology (31 vs. 10, with a *p* value of 0.045). The mean age for children with respiratory tract infections was 42.45 months (with a *p* value of 0.032), and for those with associated GERD the mean age was almost the same; 41.73 months (with a *p* value of 0.040).

All 41 patients with GERD were counseled regarding postural and feeding changes based on each individual’s age and treatment with a proton pump inhibitor—omeprazole or esomeprazole—in appropriate dose was initiated (1 mg/kg/day for children between 1 month to 1 year, 20 mg/day for children with a weight below 55 kg and 40 mg/day for those weighing over 55 kg). After 8 weeks of therapy, from a total of 41 patients, 8 (19.51%) screened positive for GERD, with a positive Boix-Ochoa score greater than 11.99, while the other 33 tested negative for GERD after control pH-metry.

## 4. Discussion

Our study aimed to evaluate the relationship between GERD and recurrent episodes of respiratory infections in pediatric patients. Although we had a small number of cases, only 53 children identified with recurrent respiratory infections (a number that did not allow us to extract many statistical data) and we found that 41 of them (77.35%) were also associated with GERD. From these 41 cases, 27 had a history of recurrent ear-nose-throat infections, while 14 had recurrent lower respiratory tract infections; regarding gender distribution, we observed that these two pathologies were more prevalent among males (31 out of 41 cases). After the 2 months of treatment with PPIs, 33 patients had a Boix-Ochoa score lower than 11.99. At evaluation one month after the end of the treatment, the respiratory symptoms (cough, sneezing, hoarseness, sore throat, rhinorrhea) had improved.

There are two main proposed mechanisms through which acidic reflux determines respiratory symptoms. The first would be an indirect one in which receptors in the esophageal wall that are sensitive to acidic pH are stimulated; this mechanism is suspected of being mediated by the vagal nerve. By stimulating the vagal fibers present in the respiratory tract, a series of inflammation-promoting substances are released, such as tachykinins, neurokinins, P substance and TNF alpha [21]. These pro-inflammatory mediators would then set off an inflammatory reaction and bronchoconstriction. Another hypothesis related to the nervous stimulation would be that the esophagus and airways have numerous transient receptor potential channels that are activated by reflux, thus leading to inflammation and increased bronchial reaction. The second proposed mechanism would be that of the direct action of acidic gastric content on airways and lung when being aspired; if micro-aspiration occurs then the usual manifestations are chronic cough and if there is macro-aspiration then repeated episodes of chemical pneumonitis or aspiration-related bacterial pneumonia appear [22].

There is always a possibility that GERD may manifest only through respiratory symptoms such as chronic cough or wheezing; this form of disease for which typical symptoms are regurgitation and/or vomiting is called “silent reflux”. This type of gastroesophageal reflux disease suggests that the degree of severity of reflux is not necessarily correlated with the appearance of respiratory symptoms, as can be demonstrated by studies on groups of children diagnosed with GERD through continuous monitoring with pH-metry. Therefore, whether the refluxed liquid is located in the proximal or distal esophagus, acid reflux plays a role in the occurrence of respiratory disorders; not necessarily through the intensity of the reflux, but through the exposure of the respiratory tissue to both the acidic environment and nonacid components provided by the refluxed liquid [23,24,25].

A study published in 2022 evaluated the type of symptoms found in 243 eligible patients with GERD, aged 14 to 88, who had pulmonary micro-aspiration diagnosed through reflux micro-aspiration scintigraphy technique, a procedure that can detect even silent micro-aspiration. The results evidenced that the most frequent symptoms were regurgitation, cough and heartburn, while the most encountered combinations were heartburn and regurgitation followed closely by cough and throat clearing. There were also patients that associated three symptoms; heartburn with regurgitation and throat clearing being present in most cases, followed by cough, regurgitation and throat clearing. These findings suggest that cough that appears in a patient with regurgitation or heartburn should guide us to investigate a possible reflux with subsequent micro-aspiration in order to prevent pulmonary damage and further bacterial infection [26].

Results from a study conducted on 65 children aged up to 12 years old with gastroesophageal reflux concluded that different respiratory manifestations in various proportions were present. In descending order of prevalence, the presence of recurrent bronchopneumonia was seen in 79.1% of cases with GERD, cough was positive in 58.3%, and the same percent was seen in patients that had nasal obstruction; 45.8% had rhinosinusitis, 61.6% had tonsillitis, 16.7% presented with asthma, and pharyngitis occurred in 12.5% of cases [27].

Also, a study conducted by a research group in New Delhi, India, on 312 pediatric patients aged 4 months to 11 years, aimed to determine the correlation between recurrent lower respiratory tract infections and silent gastroesophageal reflux using gastroesophageal scintigraphy to diagnose reflux. The results showed that more than a quarter of patients enrolled (34.6%) presented with GERD, highlighting that silent GER has an important prevalence rate, even in children older than 18 months where GER becomes pathologic [28].

Evidence exists for gastroesophageal reflux acting as an etiological factor for otitis media as gastric pepsin was identified in the middle ear fluid. This event determines mucosal inflammation through proteolytic action of pepsin over the eustachian tube cells. Also, a pH lower than 4, as happens in pathologic gastroesophageal reflux, determines ciliostasis which prevents effective clearance, thus favoring the proliferation of pathogens. A study about the clinical implications of the presence of pepsin in middle ear fluid of children with otitis media concluded that 31% of children under the age of 1 had pepsin detected in their ear, this group being definitely larger than other age groups included in the study, suggesting that pepsin from gastroesophageal reflux is a co-factor in the pathogenesis of otitis [29].

A study conducted in Egypt by a research group that aimed to identify the risk factors for recurrent otitis media with effusion (OME) among a group of 2003 children, found that, from 310 patients diagnosed with OME, 66 were associated with gastroesophageal reflux. From the 66 cases with OME and GER, 41 were recorded in children under the age of 6. Gastroesophageal reflux was the fifth risk factor as the number of cases (66) from a list of ten evaluated in the study, close to presence of nasal polyps (68), sinusitis (72) and adenoid hypertrophy (73) or allergic rhinitis (73). Thus, the study highlighted the fact that GER is an important risk factor in the repeated occurrence of otitis media with effusion [30].

In another study on pediatric patients aged 5 to 12 years old, which aimed to establish the relationship between OME and GERD, the results showed that 58% of the cases enrolled associated GERD and OME, suggesting that GERD may have an important role in the etiology of otitis media with effusion [31].

Regarding rhinosinusal pathology, in several studies GERD was found to have a high prevalence especially in cases of chronic rhinosinusitis refractory to treatment. After testing positive for presence of acid reflux in nasal cavity and then giving antireflux therapy, results showed that chronic rhinosinusitis improved, with better results in children than in adults [32].

Moving on to the next anatomical site where frequent respiratory infections in children are located, the tonsils, research data showed that, in addition to the already established mechanism by which tonsillitis occurs, gastroesophageal reflux would also play a role in its etiopathogenesis. It has been demonstrated that tonsil hypertrophy occurs when lymphocytes are stimulated due to bacterial infection but then the hypothesis of reflux mediated tonsillitis emerged. Considering this aspect, an in vitro study using human tonsillar tissue showed that when exposed to pepsin activity, the cells expressed higher levels of IL-2 and IFN-gamma, these being cytokines involved in CD4 lymphocyte proliferation that further led to tonsil hypertrophy. A particular aspect found in this study was that the phenomenon mentioned above, where pepsin acts as an antigen was observed only in pediatric patients and not in adults with tonsillitis [33].

In addition to pepsin, which acts as a trigger for the inflammatory process in the tonsils, other substances from the refluxed fluid can also play a role in the various forms of respiratory tract damage, these being hydrochloric acid, bile and trypsin. Hydrochloric acid exhibits its effects on carbonic anhydrase III, (CA III), E-cadherin and laryngeal H+/K+-ATPase, leading to several processes that ensure the negative impact of reflux over the laryngopharyngeal tissue. Thus, when CA III is missing, the bicarbonate secretion is imbalanced, leading to a pH dysregulation. Next, the reduced expression of E-cadherin, leads to the loss of cell junctions that result in an alteration of the local cell barrier with increased intercellular permeability and further deterioration of cells from the pharyngeal and nasal mucosa. Last but not least, the H+/K+-ATPase, which is found both in the stomach and larynx, as studies have shown, has been found to have a higher rate of expression in neoplastic pharyngeal cells. Bile also plays a role in the occurrence of laryngeal cancer, but this phenomenon is not the purpose of our research. Last but not least, trypsin has been shown to increase pulmonary injury and to contribute to the dentine erosion process [34].

We must mention that even a respiratory infection can be the cause of GERD aggravation, with further pulmonary aspiration and recurrent pulmonary infection. This affirmation is supported by a recent study published in 2022 that presented the role of pertussis in the exacerbation of GERD events in a group of 208 patients aged 16 to 85 years. For inclusion in the study, their profile included GERD exacerbation episodes refractory to treatment and a positive diagnosis of pertussis, resulting in 103 post-pertussis patients and 105 non-pertussis patients. They were further evaluated for presence of laryngopharyngeal reflux and aspiration by SPECT/CT. The results showed that the recent pertussis infection group had higher rates of reflux. The mechanism involved in this case is represented by a change in the thoraco-abdominal pressures due to chronic cough, this leading to GERD, hiatus hernia development and pulmonary micro-aspiration episodes [35].

In relation to the presence of recurrent pneumonia and GERD, a study published in 2021 on the risk factors of recurrent pneumonia encountered in a group of 763 patients with pneumonia, from which 87 had more than 2 episodes in a single year, found that the presence of gastroesophageal reflux was one of the factors that predisposed to repeated episodes of respiratory disease [36]. The mechanism by which this occurs is as discussed above.

Another infectious pathological entity that has a preferred pulmonary location is tuberculosis. In order to explore the impact of GERD on the risk of tuberculosis, a nationwide cohort study was conducted in Taiwan exploring patients diagnosed with GERD over a period of nine years. The results of the study showed that the presence of GERD represented an independent risk factor for pulmonary tuberculosis [37]. Considering that at global level and in our country in particular, the prevalence of tuberculosis is still high, even among children, we consider that this aspect is one that we must take into account when we are in front of a child with GERD, to whom other factors with known role in determining tuberculosis are added.

Considering diagnosis methods in GERD manifesting with respiratory symptoms, a study conducted on 515 adults with GERD diagnosed through pH-metry and who were also investigated by upper digestive endoscopy, concluded that patients who presented esophageal lesions detected by endoscopy were associated more frequently with episodes of respiratory manifestations, including infectious ones that affected their quality of life [38]. Even though it implied adult patients, the study emphasizes the important role of upper digestive endoscopy in the diagnostic algorithm of GERD and also encourages its use in pediatric reflux cases that associate important respiratory symptoms or recurrent episodes of airway infections in order to determine the degree to which reflux is involved in their occurrence, in the same manner observed in the mentioned study carried out in adults.

Regarding the treatment options in GERD, recent guidelines recommend that before initiating drug treatment, changes in lifestyle and diet must be implemented, especially in the case of infants and young children, the ages at which most presentations to the doctor due to the suspicion of GERD are registered. Therefore, initially it is recommended to use thickened or extensively hydrolyzed milk formulas for a period of 2 to 4 weeks (where there is the case to rule out a possible cow’s milk proteins allergy). In parallel with these dietary changes, it is indicated that meals should be small and frequent, the child should be kept in a vertical position for at least 30 min after each meal, the last meal of the day should be eaten 2 h before bedtime, the position during sleep should be 30 degrees from the horizontal, infants should be placed on their left side during sleep (and during the day for those that do not sit on their own yet) [1,39].

If these general and dietary measures fail, anti-secretory treatment can be initiated. The mechanisms by which the antireflux medications act in treating GERD are that of inhibiting gastric acid secretion and of reducing inflammation through their action on neutrophils, where they inhibit the production of reactive oxygen species [40].

A study presenting the GERD implication in respiratory events (recurrent pneumonia, bronchial asthma, chronic cough, chronic nasal obstruction, recurrent acute middle ear infection, recurrent tonsillitis) performed on a group of 45 children aged 3 months to 12 years, showed that antireflux treatment combined with antiallergic or surgical treatment resulted in the improvement of respiratory pathology in various degrees, suggesting that antireflux therapy has an important role in the management scheme, but does not help if used alone; other treatment methods being necessary depending on the pathology [41].

In 2022, a narrative review revised studies in which the efficacy of proton pump inhibitors and histamine H2 receptor antagonists as treatment options for pediatric GERD was tested. There were studies that evaluated only children under the age of 1 year, studies that exclusively enrolled pediatric patients aged 1 year and older and studies that included children of all ages. While the results showed that PPIs are the treatment of choice in pediatric GERD for patients aged 1 year and up, it did not find them useful in treating children under the age of 1 year that presented with unspecific symptoms such as crying, irritability, or apnea, particularly when they had no proven esophagitis or complications due to gastroesophageal reflux. Also, it concluded that there is no strong evidence in treating with PPIs asthma symptoms in children with GERD as they might actually be caused by the quantity of refluxed fluid and not by its quality, i.e., by its pH [42].

Our study showed that proper treatment with PPIs for two months lead to the resolution of GERD which was diagnosed through a Boix-Ochoa score lower than 11.99 after pH-metry. These results advocate for the effective detection and treatment of GERD in order to remove it from the equation as a risk factor for recurrent respiratory tract infections. Our results regarding the efficient use of PPIs are supported by those in the specialized literature [43,44].

Maybe the pH is not a direct trigger factor for respiratory symptoms in children compared to adults, due to the short time of exposure to the effects of acid on the respiratory tract, but reflux episodes definitely play a role in the occurrence of respiratory manifestations, not only of asthmatic ones but also of infectious ones.

Last but not least, one must bear in mind that although for the practitioner the diagnosis of GERD or silent reflux is frequently encountered in current practice and he knows that in most cases the symptoms improve as the child reaches the age of 1 year, for parents this diagnosis or the uncertainty of its confirmation is a cause of anxiety and depression, especially for young mothers who are usually the main caregivers for children particularly those under the age of 2, in which reflux symptoms (both esophageal and extraesophageal ones) are a frequent cause of hospitalization [45]. Parental mental health is an important aspect as it always influences a child’s mental and physical development.

Our study had certain limitations. First, the included lot was small and did not allow us to obtain statistically relevant data, according to a value of *p* = 0.0470. Second, pH-metry measurement is an invasive procedure, therefore it is more difficult to perform in pediatric patients, especially small ones. Also, the technique is performed exclusively in hospital, which does not respect the child’s regular schedule, pattern of meals, or daily routine. But, despite these impediments, we consider that our study contributes one more step to the attempt to establish to what extent GERD contributes as a trigger for respiratory tract infections or causes the occurrence of repeated episodes by creating an irritating and at the same time favorable environment for microbial agents.

## 5. Conclusions

Gastroesophageal reflux and respiratory infections are two of the most frequent reasons for pediatric patients to be consulted by a doctor. When respiratory symptoms do not improve under appropriate treatment, persist or infections are frequent, even in the absence of typical symptoms of reflux, the clinician should consider its presence as a source of the unfavorable evolution.

Although in our study the results did not register statistical significance, according to a value of *p* = 0.0470, the fact that approximately a quarter of the children examined in the study had repeated episodes of respiratory infections, and of these more than three-quarters also had associated GERD, shows that reflux is an important factor that contributes to airway inflammation and creates a favorable environment for repeated infectious episodes.

## Figures and Tables

**Table 1:** Inclusion and exclusion criteria for the study.

Inclusion Criteria	Exclusion Criteria
Regurgitation/vomiting unrelated to other pathology	Treatment with proton pump inhibitors over the last 3 months
Poor weight gain/ weight loss in infants and small children	Treatment with aspirin or other non-steroidal anti-inflammatory drugs
Protracted crying in infants	Treatment for *H. pylori* infection
Chronic cough that did not improve under treatment	Foreign body aspiration
Cough that occurred at night time	Systemic diseases that caused esophageal lesions
Diagnosis of recurrent upper respiratory tract infection (sinusitis, pharyngitis, laryngitis, rhinitis, tonsillitis, acute otitis media)	Known diagnosis of bronchopulmonary dysplasia, primary ciliary dyskinesia, cystic fibrosis, sleep apnea, asthma
Diagnosis of recurrent lower respiratory tract infection (pneumonia, bronchiolitis)	Gastrointestinal blood loss identified at endoscopic examination
	Surgery for esophageal or gastric pathology
	Known food allergy
	Cardiac abnormalities

**Table 2:** Distribution of cases regarding respiratory tract infection and GERD presence.

		Gastroesophageal Reflux		Total
		Negative	Positive	
Recurrent respiratory tract infections	Upper tract	8	27	35
	Lower tract	4	14	18
Total		12	41	53

**Table 3:** Data on Gender Distribution and Place of living among patients.

Cases	Gender		Place of Living	
	Male	Female	Urban	Rural
With recurrent respiratory tract infections	39	14	40	13
With recurrent respiratory tract infections and GERD	31	10	24	17

**Table 4:** Gender distribution according to the type of cases included in the study.

	Gender		Total
	Male (% of Total)	Female (% of Total)	
Cases with RURTI * without GERD	5 (9.43)	3 (5.66)	8 (15.09)
Cases with RURTI and GERD	20 (37.73)	7 (13.20)	27 (50.94)
Cases with RLRTI ** without GERD	3 (5.66)	1 (1.88)	4 (7.54)
Cases with RLRTI and GERD	10 (18.86)	4 (7.54)	14 (26.41)
Total	39 (73.58)	14 (26.41)	53 (100)

* RURTI = recurrent upper respiratory tract infections. ** RLRTI = recurrent lower respiratory tract infections.

## Data Availability

Data available on request from the corresponding author.
